# Extracting Labeled Topological Patterns from Samples of Networks

**DOI:** 10.1371/journal.pone.0070497

**Published:** 2013-08-12

**Authors:** Christoph Schmidt, Thomas Weiss, Thomas Lehmann, Herbert Witte, Lutz Leistritz

**Affiliations:** 1 Bernstein Group for Computational Neuroscience Jena, Institute of Medical Statistics, Computer Sciences and Documentation, Jena University Hospital, Friedrich Schiller University Jena, Jena, Germany; 2 Department of Biological and Clinical Psychology, Friedrich Schiller University Jena, Jena, Germany; Technical University of Madrid, Italy

## Abstract

An advanced graph theoretical approach is introduced that enables a higher level of functional interpretation of samples of directed networks with identical fixed pairwise different vertex labels that are drawn from a particular population. Compared to the analysis of single networks, their investigation promises to yield more detailed information about the represented system. Often patterns of directed edges in sample element networks are too intractable for a direct evaluation and interpretation. The new approach addresses the problem of simplifying topological information and characterizes such a sample of networks by finding its locatable characteristic topological patterns. These patterns, essentially sample-specific network motifs with vertex labeling, might represent the essence of the intricate topological information contained in all sample element networks and provides as well a means of differentiating network samples. Central to the accurateness of this approach is the null model and its properties, which is needed to assign significance to topological patterns. As a proof of principle the proposed approach has been applied to the analysis of networks that represent brain connectivity before and during painful stimulation in patients with major depression and in healthy subjects. The accomplished reduction of topological information enables a cautious functional interpretation of the altered neuronal processing of pain in both groups.

## Introduction

Concepts from network theory have successfully provided a general framework with wide applicability to discover differences as well as similarities in the structure and function of systems and to understand organizational principles that drive interacting elements. In particular the neurosciences have benefited from the progress in modern network theory [1]. The organization of brain areas has been studied empirically [2], [3], [4], [5] 7]and theoretically [6], [7]. It has been shown that pathologically altered neural network topology and altered functional connectivity seem to be correlated with cognitive and psychiatric disorders such as Alzheimer's disease [8] and schizophrenia [9], [10] that are described as disconnection syndromes [11], [12], [13] or epilepsy [14]. In this context network measures enable quantification of pathological abnormalities in brain networks. For an overview of network measures that quantify global and local network topology we refer e.g. to [15], [16], [17].

System analysis is most commonly performed on single networks, although the analysis of single networks can be problematic as one network represents only an instance of the system at hand and is not fully representative of the system itself. With variance in the shape and properties of such instances, a system's underlying properties and phenomena cannot be easily captured in their entirety in one single network alone. Such a variance might for example be displayed in fluctuations in the distribution of links or connection strengths. In this work we investigate samples of networks drawn from a specific population. An investigation of samples of networks can offer further advantages besides avoiding the drawbacks outlined above, i.e. the incorporation of pairwise different vertex labels into the analysis. Vertex labeling exists in networks from many domains but is particularly prevalent in neuroscience and should be considered. Within this framework we investigate samples of associated directed networks of equal size whose vertex labeling comprises functional relevant information on the location of each vertex in the network. The investigation thereby focuses on the extraction of characteristic topological patterns shared by sample element networks, and less important topological patterns are filtered out. It is based on network motif detection in single networks without vertex labeling [18], [19]. Network motifs constitute an interesting property of local network topology: They are small, conserved and overrepresented directed subnetworks (or subgraphs), which potentially act as building blocks or as elementary information processing circuits and thus may make important contributions to the functionality of their network. In this context it is assumed that individual real-world networks (or classes of networks) possess characteristic combinations of network motifs that reflect topological constraints related to the functionality of the represented system and its history of development [18], [19], [20], [21]. Network motif detection has been applied to decompose various single real-world networks, e.g. protein-protein interactions, electronic circuits, the World Wide Web, transcriptional regulatory networks, synaptic connections in neural assemblies and connections within and between cortical areas [18], [19], [20], [22], [23], [24]. A variation of network motif detection was presented in [24] and in [23] where structural motifs with contained functional motifs and composite network motifs were detected, respectively. Network motif detection may also be generalized to the case of weighted networks [25].

The conventional workflow to detect motifs in a single network involves three fundamental steps that are computationally expensive: (1) Enumeration [18] or sampling [26], [27] of subnetworks that are induced by a vertex set of 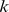 vertices. (2) Partitioning of these subnetworks into topological equivalence classes and obtaining their counts, i.e. determining graph isomorphism for the subnetworks. (Subnetworks 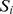 and 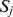 are isomorphic if they contain the same number of vertices connected in the same way. This might only be apparent after a permutation 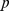 of their vertices 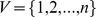 such that 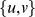 is an edge of 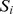 if and only if 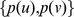 is an edge of 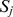.) (3) Determination of statistical significance of subnetwork counts.

Our approach differs from the conventional workflow outlined above in a few ways. These differences stem from the subnetwork enumeration in all sample element networks and an accordingly adjusted statistical test to assign significance to subnetwork counts obtained over the sample. Moreover, the enumeration must account for the vertex labels. This novel approach complements and expands an analytical approach we reported on previously [28].

This publication is subdivided into parts as follows. In Section 2 a general description of our approach to extract topological patterns from network samples is provided. Properties of the null model we used for the statistical test are emphasized and a means to estimate its parameters is proposed. We apply this approach to EEG data. The samples of networks are derived from connectivity data obtained from EEG recordings of patients with major depression and healthy controls before pain perception and during pain processing [29]. Analysis of characteristic topological patterns in these network samples has clinical relevance since the understanding of the connection between pain and depression is preliminary. Section 3 comprises a concise summary of resulting data; for further detail on data used in the study, its acquisition and pre-processing, the Materials S1 is included. A discussion and interpretation of the results with regard to altered effective connectivity patterns in patients with major depression and the control group is provided in Section 4. We conclude in Section 5 with a general discussion of our approach in the context of the most notable findings from the current literature.

## Methods

### Ethics Statement

Prior to the experiment detailed information on the aim and the procedures of the experiment was provided to each subject and written informed consent was obtained. The procedure was approved by the Ethics Committee of the Friedrich Schiller University (reference number 2282–04/08).

### Methodology

**Figure 1:**
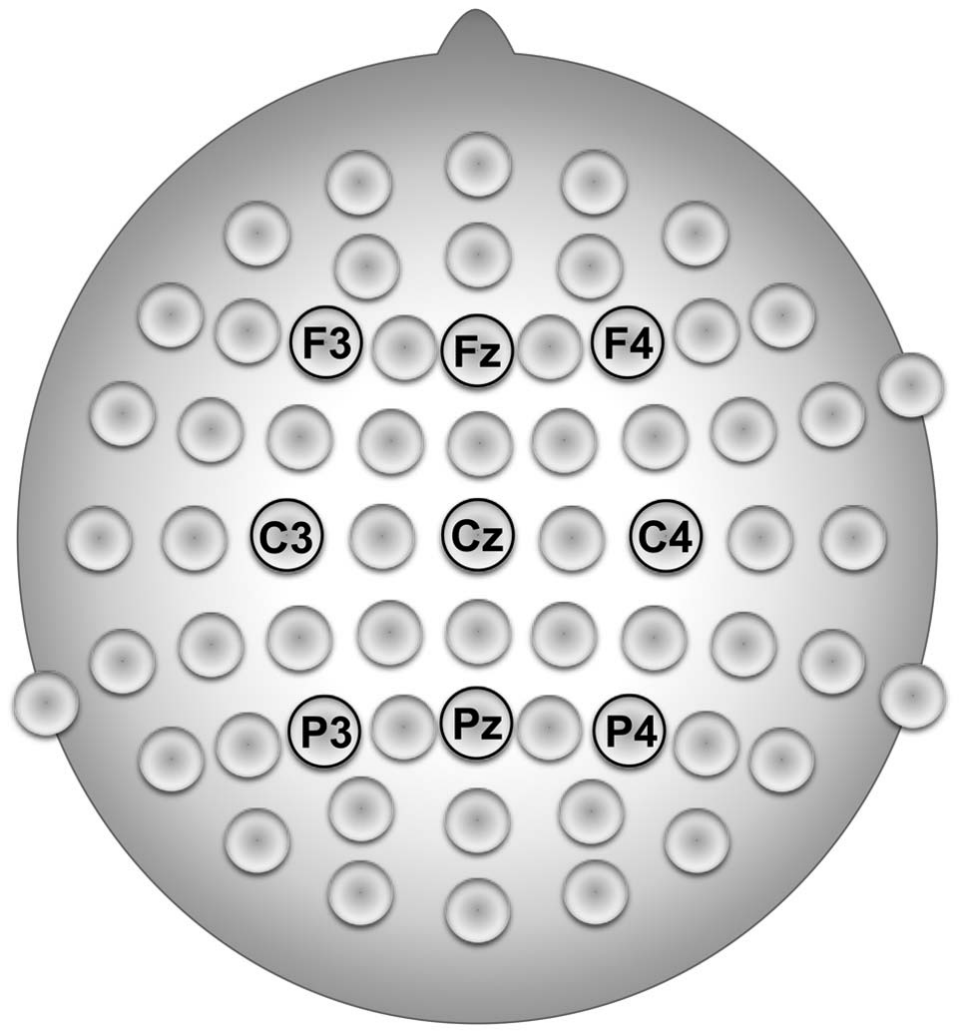
The location of the nine EEG electrodes selected for the connectivity analysis according to the extended International 10–20 System of Electrode Placement. The vertex labeling of the networks that represent significant interactions entails the information about these electrode locations.

Given a sample of directed networks of equal size and with the same pairwise different vertex labeling, the appearance of induced connected and directed vertex labeled subnetworks (subgraphs) is analyzed with respect to overrepresentation. A possible overrepresentation of subnetworks is established by a comparison of their counts obtained from the sample to the respective counts in random networks that are specified according to a suitable null model. Subnetworks that are shared significantly often by sample element networks can be interpreted as characteristic topological patterns (sample-specific network motifs with fixed pairwise different vertex labeling). These are, by definition, important topological patterns whose further investigation promises to add insight into the phenomena that underlie the network structure. Moreover, they allow for comparing and distinguishing network samples on the basis of topological properties and might reveal differences in connectivity. Characteristic topological patterns might not be exclusively constructed of edges that occur frequently in the sample but might also contain less frequent edges. Subnetworks that are not overrepresented in the network sample are treated as less important and thus are discarded in a subsequent interpretation of network structure-function relationships. The location of a characteristic topological pattern in a network clearly is relevant for the interpretation of its function in the context of the network, i.e. this kind of spatial information is linked to an underlying process or a property. The subnetworks of EEG-derived networks that we analyze in Section 2.6 are locatable in the sense that they have an unambiguously identifiable position in their network, which is a direct consequence of the pairwise different vertex labeling where each vertex label corresponds to exactly one EEG electrode location in the 10–20 system (Figure 1). The presented method can be applied to networks with any pairwise different vertex labeling that entails different functional information and makes all vertices distinguishable and each edge unique. Due to its importance for functional interpretation the information on vertex labeling is preserved. Pairwise different vertex labeling has important consequences for the investigation of topological patterns: the respective network samples do not contain isomorphic subnetworks and each subnetwork occurs at most only once in a single network. This differs from original network motif detection in single networks without vertex labeling. Instead of determining graph isomorphism, we can directly compare two subnetworks. They are identical if and only if they share exactly the same set of edges (as opposed to only sharing their patterns of interconnections), i.e. their adjacency matrices are identical. Accordingly, step (2) of the conventional workflow for detecting network motifs in the original sense is not required, which is an advantage with respect to the computational complexity of our task. The consequence for statistical analyses is that it's not possible to assign significance to subnetwork counts if the sample size is too small. Extracting locatable characteristic topological patterns from a network sample begins with an exhaustive enumeration of subnetworks that are induced by 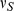 different vertices in every sample element network. It is followed by an appropriate statistical test to assign significance to their counts. Notably, even for subnetwork size 2 the set of characteristic topological patterns yielded by the statistical test of our approach might be different from a set of subnetworks of the same size yielded by applying a preset threshold to their counts obtained over the sample.

### Exhaustive enumeration of subnetworks

Let 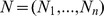 be a sample of networks 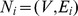 that all comprise the same set 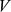 of 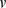 vertices with pairwise different labels and a specific finite set 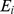 of directed edges. In particular, all sample element networks 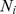 are required to have an identical pairwise different vertex labeling. The ordered pair 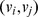 denotes a directed edge (arc) that leaves vertex 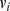 and connects to vertex 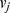. The vertex 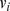 is called the tail and vertex 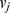 the head of the edge. All networks of the sample 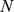 are simple graphs. Consequently, they do not contain loops (edges where tail and head coincide) or multiple edges (multi-edges – edges that have the same tail and the same head).

Whereas original network motif detection usually chooses 3, 4 or 5 as values for the subnetwork size parameter 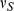, in our approach, due to the preserved location information of the subnetworks, it makes sense to detect topological patterns even of size 2. Generally, here and in original network motif detection, 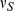 is assigned a small value to avoid the long computation times as well as difficulties in assessing functional roles of significant patterns. Exhaustive enumeration of induced subnetworks in all element networks of a network sample is practical for many network sizes, especially those that are encountered in analysis of EEG recordings (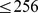 vertices). Subnetwork enumeration in 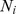 is performed by investigating all combinations of 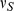 labeled vertices to determine whether they induce a connected subnetwork in 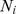. Thereby, the count of every such vertex-labeled directed subnetwork in the sample 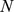 is obtained. Alternatively, for larger network sizes and lower edge densities, where many vertex combinations might not form subnetworks, another enumeration scheme might be better suited. For example an enumeration scheme based on the ESU algorithm [27] might be faster and more memory efficient for those networks as it recursively finds for every vertex the set of vertices that can be used to extend the subnetwork rooted at it. However, for our data that is composed of dense and smaller networks and for the investigated small subnetwork sizes we found that investigating all vertex combinations is faster (using the Matlab programming language).

At this point it is still unknown which subnetworks constitute characteristic topological patterns of the sample 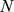. To shed light on this, these counts are compared to respective counts in null model networks to assign significance to the subnetworks.

### A degree sequence preserving null model

Distinctly nonrandom characteristics in network topology are linked to functionally important substructures. A graph null model is needed to construct a reference system that contrasts such regularity with random effects that also influence the formation of the network topology. Such a null model is used for statistical testing to identify those vertex-labeled subnetworks that occur in the network sample significantly more often than in a sample of suitable random networks. A suitable null model might be based on randomization of network data and represents the null hypothesis that edges connect to vertices without preference. The architecture of the resulting randomized networks has to be formed by a random process that does not entail selection for or against particular substructures but still maintains certain characteristics of the network topology. Particularly, the effects of any process that created structures with functional relevance in the networks have to be reversed during the generation of the null model networks. Choosing a suitable null model that fits given network data is an open problem. In particular, it is difficult to decide which low-level topological properties of the network data the null model networks should capture while at the same time the connectivity between vertices varies stochastically. Using an inappropriate null model in the statistical test might introduce a bias in the assignment of significance to subnetworks [30], [31]. The null model widely employed by original network motif detection preserves the in-degree and out-degree sequence. The in-degree and out-degree sequence is a basic and important attribute of a directed network which consequently should be accounted for in the generation of reasonable null model random networks [30], [32]. This property determines the topology of a network to a certain degree by imposing constraints on potential locations of edges and therefore it ultimately affects many of the network's properties. Incorporation of the vertex degree sequence into the null model potentially yields a statistical test for significant subnetwork counts with a “good” amount of restrictiveness so that not too many false positive results nor too many false negative results are expected. The associated random networks are usually either generated by the configuration model (“stubs-pairing”) [33], [34], [35], [36], [37], [38] or by a Markov chain Monte Carlo method (“edge-switching”) [18], [34], [39], [40], [41].

By means of the “edge-switching” algorithm we generate a certain number of randomized networks for each input network 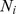 of a network sample 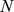. The fundamental idea of the “edge switching” algorithm is to rewire an input network by means of a series of random reconnections of edges that do not change either the in-degree or the out-degree of any vertex. For this elementary graph transformation two directed edges (v_i_,v_j_) and (v_i_',v_j_') are uniformly selected at random. The heads of both selected edges are exchanged to yield two newly rewired edges (v_i_,v_j_') and (v_i_',v_j_) if this exchange does not generate multiple edges or loops in 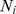. Otherwise the switch is rejected and the procedure continues to randomly select the next pair of directed edges. The attempt to switch a pair of directed edges is repeated 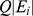 times, where 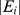 is the number of directed edges in 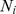 and 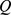 is a (“mixing”) parameter which is chosen large enough to allow the underlying Markov chain to converge to its stationary distribution. In particular, rejected switches, which correspond to the transition from a network to itself, are also counted. Note that the edge-switching algorithm does not preserve the number of bidirectional links, which might be reasonable in several contexts [31].

No a-priori bound exists for the mixing time of the underlying Markov chain and it is not known for rapidly mixing for general degree sequences, which is a clear drawback of the “edge-switching” algorithm [42]. Ideally, the choice of 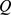 ensures that the algorithm generates with uniform probability every directed network with a prescribed degree sequence. In [34] the authors find empirically “that for many networks, values of around 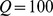 appear to be more than adequate”. However, current literature on network motif detection does not usually specify the selection of the parameter 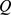 and often the choice of the number of random realizations of the investigated single network remains vague. To save computational time and main memory it is also desirable to generate only as many random realizations of the input network's in-degree and out-degree sequence as necessary to ensure that the distribution of relative subnetwork frequencies in these random networks is likely to differ only within sufficiently small bounds from a distribution obtained by generating a larger number of realizations. Generating 1000 random networks has been suggested [18], [23], [39], [43] but the authors do not explicitly outline the motivations for this choice of size for the random ensemble. In [34] between 1000 and 10000 random networks were used for network motif detection, also without further justification. Another study [32] on the correlation profile and clustering of the internet used 1000 and 100 randomized networks, respectively, without detailed explanations.

We apply simple but effective procedures to determine both quantities more accurately with regard to given network data. As already mentioned, our aim is to analyze a network sample, not single networks. Therefore, it would not be feasible to determine the value of 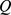 for every sample element network in a reasonable amount of computational time. Instead we propose to identify one sample element network that is representative of all other analyzed networks of this sample with respect to the in-degree and out-degree sequence (which is the property that we preserve in network randomization). The identification of the representative network might be performed by means of calculating the mean in-degree and out-degree sequence of all networks of the sample and finding the one whose degree sequence has minimal distance to it, according to the metric induced by the maximum norm. This network can subsequently be used for determination of the parameter 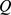 that is needed for the generation of null model networks for each sample element network.

We are interested in the value of the mixing parameter 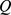 for which the edge-switching algorithm uniformly samples networks with prescribed in-degree and out-degree sequence. Several values of 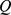 are thereby analyzed. Then the performance index 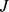 is used, similar to the test statistics of the chi-squared goodness-of-fit test, to investigate the influence of 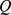, because it cannot be ensured a priori that the performance index 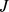 is asymptotically chi-squared distributed. Given 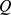, by means of 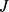 it can be investigated whether every network with the prescribed degree sequence of the representative network will be generated with equal probability. Each unique network with the prescribed degree sequence corresponds to one category in this test. To approximate the value of the common probability 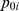 for each category under the uniform distribution hypothesis for generating each network (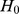) at a minimum a good lower bound for the number of networks that have the same degree sequence as the representative network has to be known. For this, large numbers of random realizations of the representative network have to be generated using the edge-switching algorithm with different values of 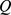. The results of the independent simulations must then be pooled together, since for different 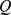 the algorithm might sample networks from different regions in the network sample space. This union set of networks with the prescribed degree sequence can be used to determine a lower bound for the number of pairwise different networks. Because this procedure provides only a lower bound, one is interested in obtaining samples as large as possible, especially for larger values of 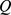. It is to be expected that network generation with very distinct values of 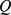, e.g. a small value and a large value will probably result in almost entirely different sets of generated networks with only few networks being shared among the sets. Unfortunately, these computations will eventually be constrained by time and main memory limits and one must stop the simulation at some point. These limitations are less pronounced when network sizes are comparatively small, as e.g. in EEG-derived networks that are investigated in computational neuroscience.

Now we can calculate 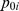 by using the determined number of pairwise different networks with the prescribed degree sequence to compute 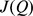 for every 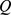. Under 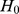 expected counts 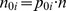 for every network should ideally be at least greater than one and often they must be greater than five when the chi-squared goodness-of-fit test is applied. Due to the aforementioned constraints on computational resources the number 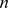 of generated networks might not be large enough to satisfy the assumption of the expected counts. A binning of networks does not make sense because there is no natural ordering. Still one is advised to calculate the performance index. As mentioned above it cannot be assumed a priori that 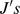 distribution under 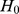 is chi-squared with 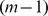 degrees of freedom, where 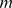 is the number of categories. In that case the corresponding quantile might be determined by means of Monte Carlo simulations. The smallest 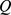 for which 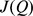 falls below the 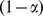-quantile is selected. Otherwise, if 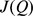 exceeds the 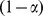-quantile of 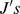 probability distribution under 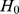 for all 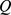 the edge-switching algorithm does not generate uniformly distributed networks for any 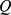. Then we suggest using that value of 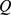 for the randomization for which 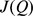 is minimal. We describe in the Materials S1 how to obtain an estimation of an upper bound for the number of networks with prescribed degree sequences of the representative network by means of an appropriate decomposition of its adjacency matrix.

After identifying the value of the mixing parameter 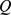, detection of the number of random realizations 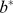 for every sample element network required for a reliable detection of characteristic topological patterns is described. We propose to generate an upper bound 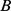 of Bootstrap network samples of size 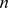 by applying the edge-switching algorithm. Based on these Bootstrap network samples, a reference distribution 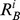 of relative subnetwork frequencies is calculated by enumeration of all interesting vertex-labeled directed subnetworks 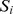. This distribution is compared to distributions 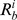 obtained in the same way from lower numbers 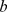 of Bootstrap network samples. 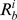 is accepted to be sufficiently close to 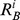 if it holds 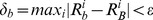 for an arbitrary fixed 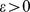. Finally, 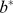 is defined by 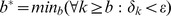.

### Assignment of significance to subnetwork counts

After generating a random network ensemble that consists of 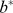 random realizations of every sample element network's degree sequence, statistical significance can be assigned to the subnetwork counts that have been obtained from the input network sample. By enumeration of these subnetworks in the random network ensemble their relative frequencies can be obtained, which in turn are needed to compute p-values for their counts in the input network sample. Since several subnetworks are tested with respect to a significant overrepresentation in the sample an alpha-adjustment has to be applied. For this, we suggest using the Bonferroni-Holm correction [44] with a multiple significance level of 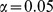 for all multiple test procedures to conservatively control the familywise error rate for all hypotheses at 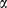 in the strong sense instead of controlling the expected proportion of incorrectly rejected null hypotheses (false discovery rate). Locatable characteristic topological patterns, that is sample-specific network motifs with pairwise different vertex labels, are those subnetworks that have significantly enlarged counts over the network sample.

### Application to EEG connectivity networks

**Figure 2:**
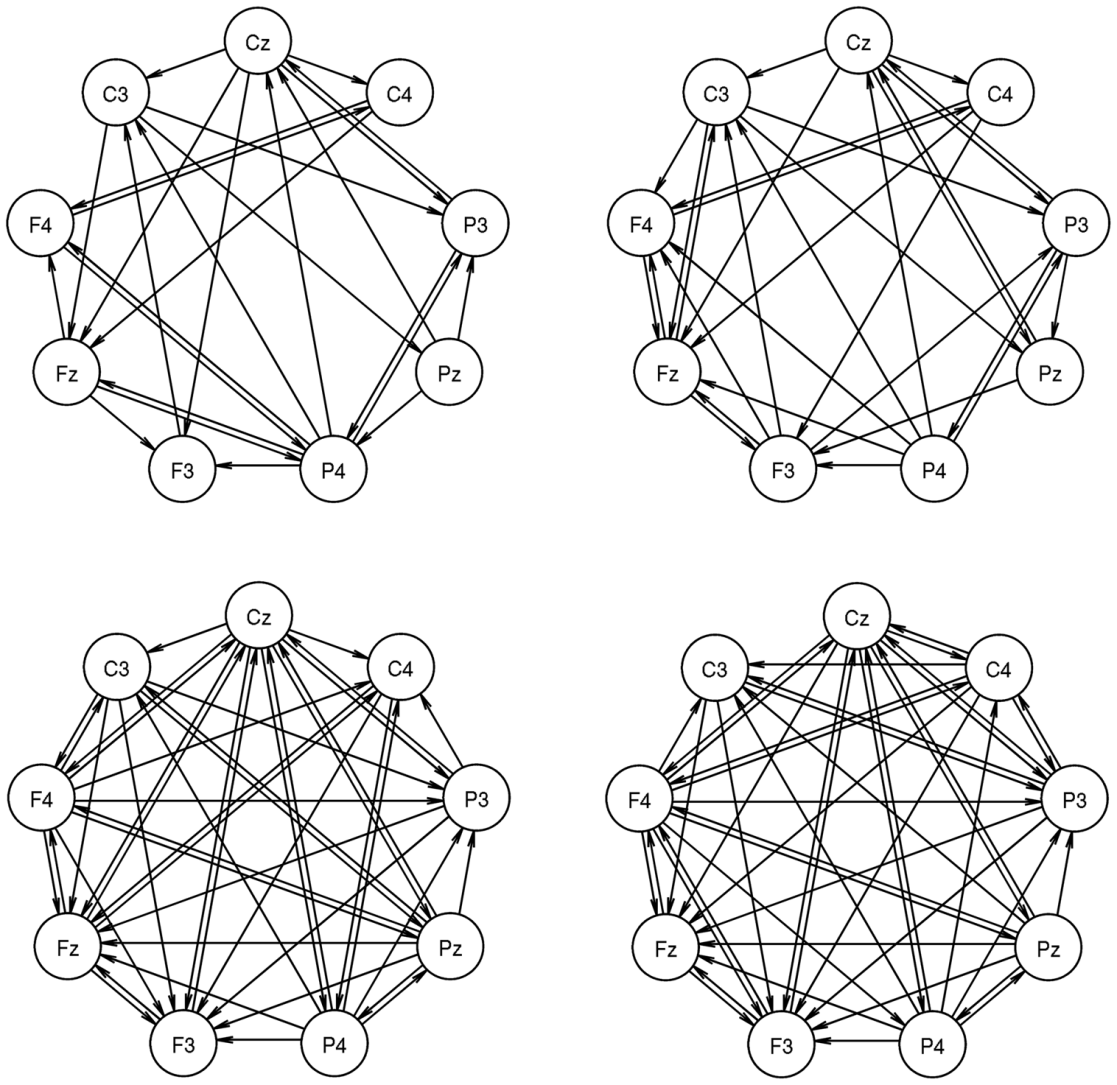
Examples of effective connectivity networks (ECNs). The upper row shows networks of a MD patient (#1) before (left network) and after (right network) left hand side stimulation. The lower row shows networks of a healthy control subject (#1) accordingly. These networks are representative for samples of equal sized networks with identical pairwise different vertex labels.

To provide a proof of principle we applied our proposed method to real-world data. The network samples are taken from a previous EEG experiment and subsequent investigation of effective connectivity [29]. They were also examined in [28], [45]. In the following we denote these networks as effective connectivity networks (ECNs). The ECNs that we examine here describe directed interactions before pain perception and during pain processing in a group of patients with major depression (MD) and a healthy control group (HC). The EEG was recorded continuously from 60 electrodes during each subject's electrical intracutaneous stimulation at the tip of the middle fingers of the right and the left hand. For the connectivity analysis data was used that was recorded from nine selected electrodes (F3, Fz, F4, C3, Cz, C4, P3, Pz and P4; re-referenced to linked ears) that are situated above brain regions associated with pain processing, attention and depression (Figure 1). To compare network topology in the pre- and post-stimulus condition, signal sections of 700 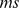 duration were extracted before and after stimulus onset. The effective connectivity between each ordered pair of these electrodes was measured by means of the generalized partial directed coherence (gPDC) [46]. One gPDC value results for each of the 72 directed interactions and the effective connectivity that we are interested in is given by significantly increased gPDC values. It is represented as an ECN, which consists of nine vertices and the significant interactions. In the context of this study the vertex labels signify the location of EEG electrodes and topological patterns might be denoted as interaction patterns. Eight network samples result from the association of the group assignment (MD or HC) to a particular combination of stimulus condition (pre- or post-stimulus) and stimulated side (left or right hand). The sample size is fifteen for the “MD – post stimulus – right hand” sample, whereas it is sixteen for all other samples. Example ECNs are depicted in Figure 2. For more detailed information on their fundamental characteristics, the EEG experiment and on the investigation of effective connectivity, we also refer to the Materials S1.

Each sample's member networks comprise unique information about the investigated underlying phenomena, namely the processing of painful stimuli in the so-called neuromatrix of pain [47] in both groups. Analyzing the interaction patterns in these samples of ECNs can contribute to a more refined understanding of the relationship between pain and depression, as many aspects of this connection remain poorly understood. Yet it is known that in many cases depression is a comorbidity of chronic pain and conversely, chronic pain is often an additional symptom of depressed patients [48], [49]. However, the physiological basis for pain perception, pain processing and the sensitivity to painful stimuli of depressed patients remain unclear. It is supposed that the processing of painful stimuli in the neuromatrix of pain is altered in depressed patients [50], which consequently is expected to be reflected in the effective connectivity being altered, too.

**Figure 3:**
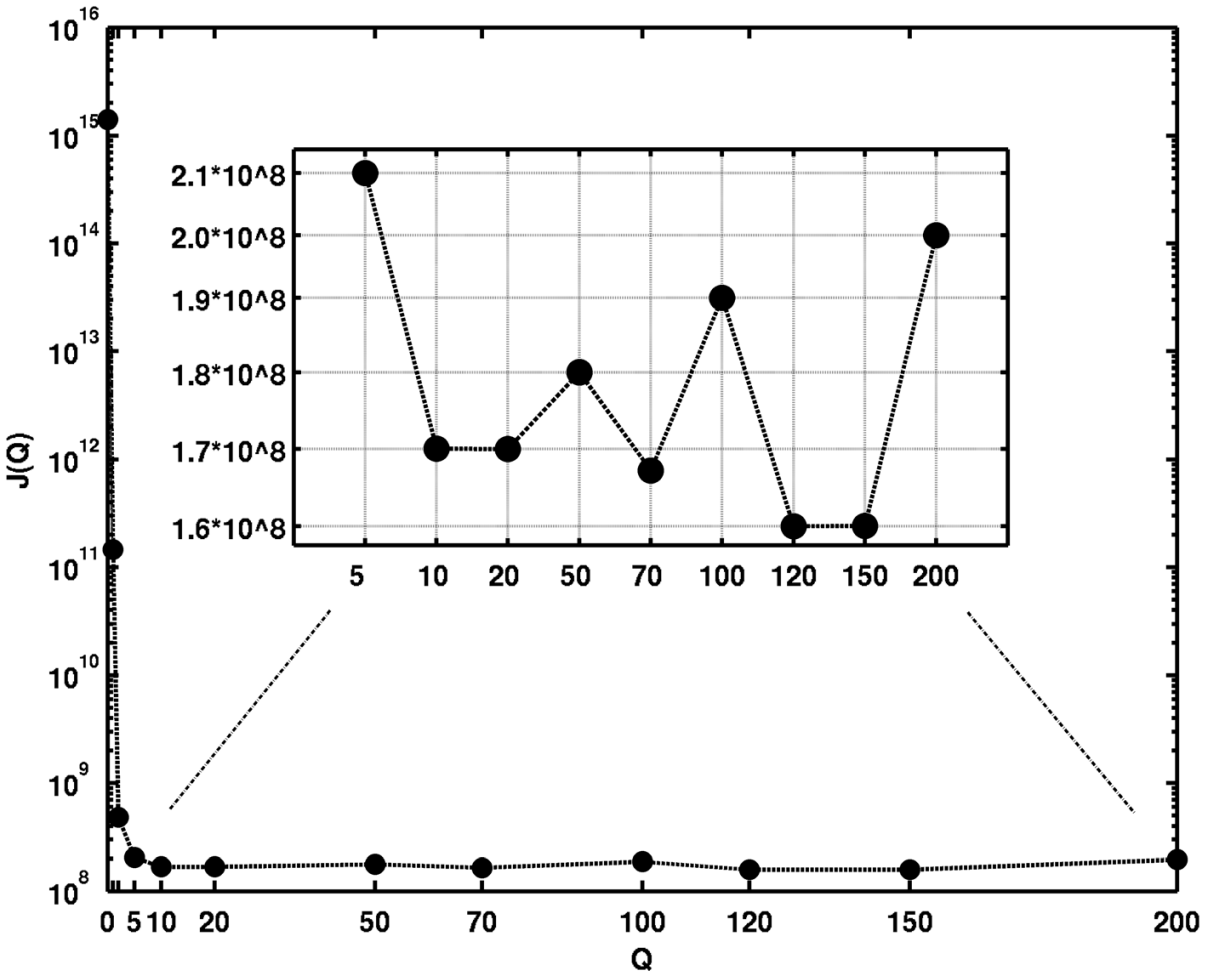
Results of the determination of the mixing parameter 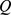 for which the edge-switching algorithm samples networks with prescribed in-degree and out-degree sequence uniformly. For every 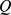 the resulting value of the performance index 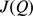 exceeds the corresponding 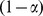-quantile of the test statistic distribution.

The method to detect locatable characteristic topological patterns was applied to each of the eight network samples separately. As shown by our previous investigations subnetworks of size 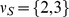 are interesting, because an interpretation of larger interaction patterns seems difficult to obtain given the current state of knowledge on pain, depression and information processing in the brain. To further reduce the complexity when comparing ECN samples, we decided not to detect and evaluate the appearance of every subnetwork of size 3, but instead we focused on those 3-subnetworks that occur in at least one sample of ECNs at least four times, because these subnetworks represent the most promising candidates for network motifs. This restriction decreases the number of potential 3-subnetworks from 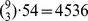 to only 134 subnetworks. The equation above accounts for 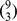 combinations of 3 different vertices where each combination might form one of 54 different connected vertex-labeled 3-subnetwork topologies. We generated null model networks by means of applying the edge-switching algorithm to all element networks of each network sample. The resulting randomization disintegrates structures with functional relevance in the interplay of anatomy and function of the underlying recorded brains. For determination of the parameter 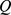 we computed a representative ECN, which turned out to be an element of the “HC-post-left” sample. Random realizations of the representative ECN were generated using the edge-switching algorithm with different values of 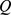. The results of the independent simulations were then pooled together to yield a total of 190,400,001 networks. In this set we found 101,996,824 pairwise different networks with the given prescribed degree sequence, which seems to be an appropriate lower bound for the number of such networks. The actual number of networks is much larger but due to time and main memory limits of the computations we had to stop the simulations at this point. During this random network generation process we observed a few interesting aspects: First, we noted that the network most often yielded from the randomization process was the input network itself. This can be explained by the incorporation of the results obtained with low values of 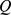, where after only few edge switch attempts (that moreover might have been unsuccessful) the input network was yielded as the output of the underlying Markov chain. Second, we noticed that generating networks with very distinct values of 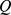 resulted in almost entirely different sets of generated networks with only few networks being shared among the sets. This observation was expected, since for small values of the mixing parameter the edge-switching algorithm can cover only a small part of the network configuration space. We then calculated 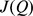 for each of the simulation results obtained by using a single value of the mixing parameter 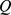. By using the lower bound for the number of pairwise different networks with the prescribed degree sequence we obtained 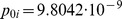 as value for the uniform probability of network generation under 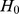. Due to the aforementioned constraints on computational resources the number of random realizations of the representative network (approx. 18,000,000) generated for every given value of 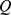 was not large enough to satisfy the assumption on the expected counts. This number was also much smaller than the lower bound on the number of pairwise different networks (101,996,824). Finally, for every 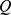 the resulting value of the performance index 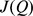 was greater than the corresponding 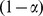-quantile of the test statistic distribution. Thus, the edge-switching algorithm seems to generate networks with ECN degree sequences with non-uniform distribution for every 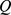. It was intriguing to uncover that the proportion of networks that were generated three times in each sample was disproportionately high. Mainly, these sample elements are responsible for increased 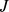-values and the large deviation from the expected 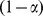-quantile. Because we could not identify a particular 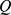-value, where 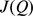 falls below the 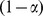-quantile, we decided to use the lowest value of 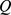 for which 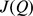 was minimal. As it turned out this was 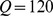 (see Figure 3). According to our outlined procedure we determined the required number of random realizations for every sample element network as 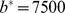.

In the following Section we present the neurophysiological results of our approach applied to these network samples.

## Results

**Figure 4:**
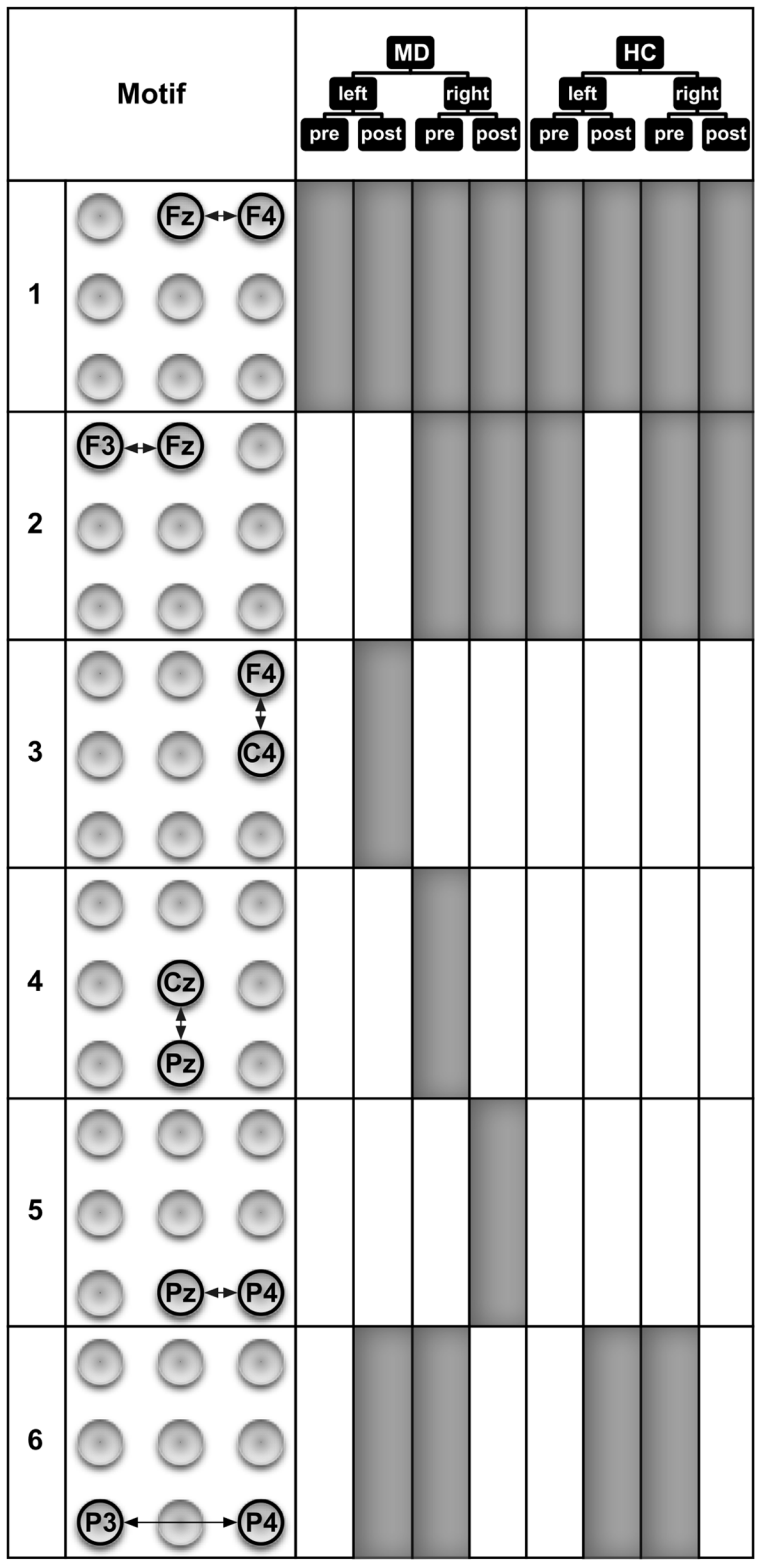
Motifs of size 2 that were detected in the eight ECN samples. The occurrence of a 2-motif in an ECN sample is indicated by filled areas. These motifs represent important patterns of directed interactions that occur before and during the processing of painful electrical stimuli. The ECN samples stem from combinations of the group assignment and experimental conditions: MD–patients with major depression, HC–healthy control subjects, left and right–stimulated sides, pre and post–time windows with respect to the stimulus condition.

Figure 4 showing motifs of size 2 revealed several interesting points. MD patients show slightly more motifs of size 2 than HC subjects. However, 8 out of 12 motifs in MD and 8 out of 9 motifs in HC are similar with respect to the motif and the time period when it occurs. Overall this demonstrates that motifs of size 2 show strong communalities in processing between the groups. This shows that the method allows the identification of robust connections. One of these functional connections is present for all time windows (Fz↔F4) for both sites of stimulation. This motif was seen in our earlier work [28]. It is likely that this connection represents a part of the background activity or attentional processes which are independent of group (MD, HC), time period (pre, post), or site of stimulation (left, right). Other motifs, e.g. F3↔Fz, are primarily found in association with the stimulation of the right hand. So this processing contralateral to the stimulation site might represent processes of preparing to and analyzing the nociceptive input. Interestingly, this motif is the only 2-motif in HC that is also not present in MD. It occurs during the pre-stimulus period prior to the left hand stimulation in HC. This activity might represent a preparation in advance of the hand stimulation, e.g. the process of distributing attentional resources. The lack of the F3↔Fz motif in MD fits with additional motifs in MD which do not appear in HC. Similarly to previous work [28] all these additional motifs are located in the right hemisphere or midline. This might reflect the role of the right hemisphere in the processing of emotions and mood in MD patients [50], [51], [52], [53], [54].

**Figure 5:**
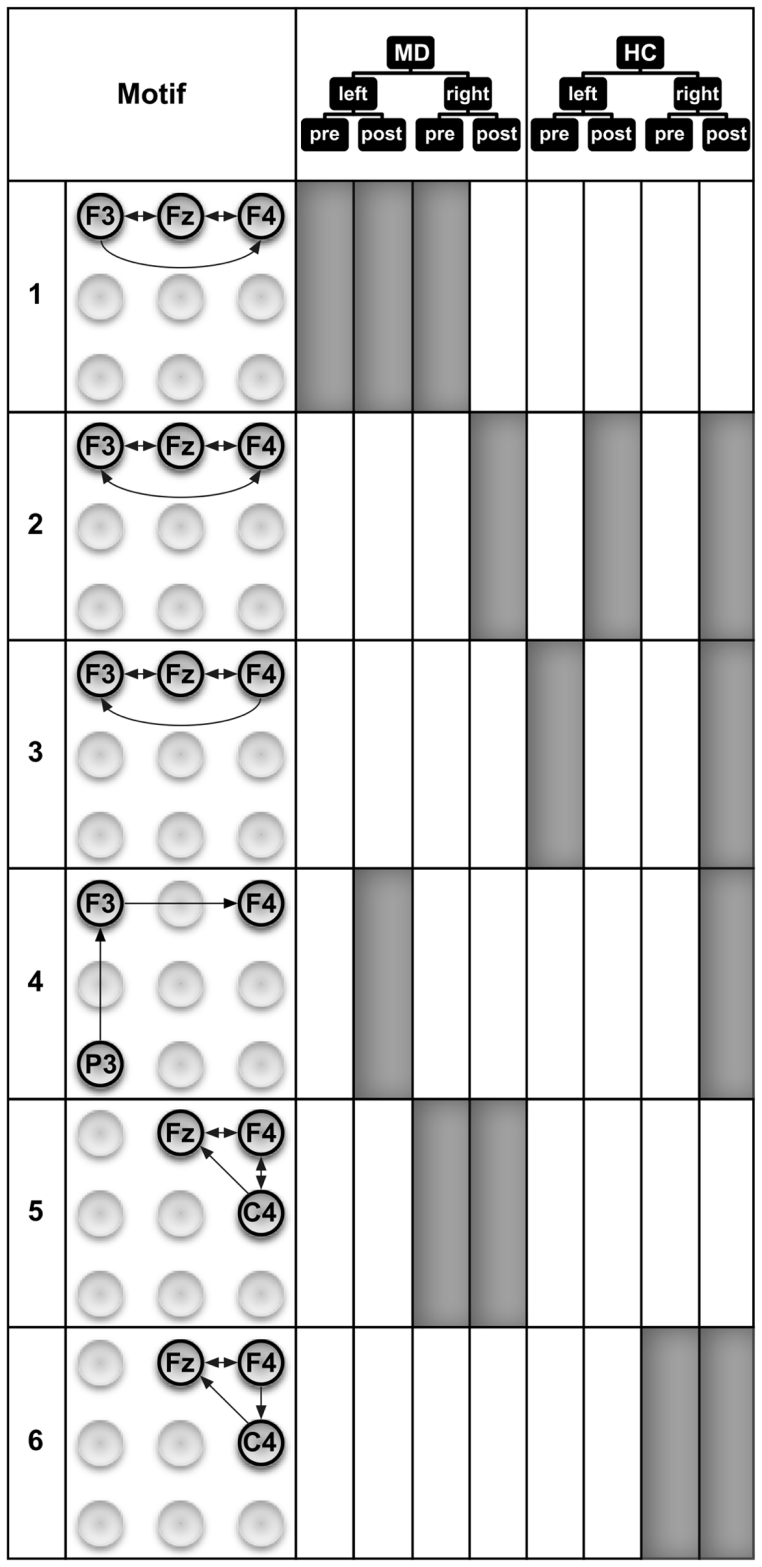
Six out of twelve motifs of size 3 that were detected in the eight ECN samples. The occurrence of a 3-motif in an ECN sample is indicated by filled areas. These motifs represent important directed interaction patterns of brain activity recorded at three different EEG electrodes that occur before and during the processing of painful electrical stimuli. The ECN samples stem from combinations of the group assignment and experimental conditions: MD–patients with major depression, HC–healthy control subjects, left and right–stimulated sides, pre and post–time windows with respect to the stimulus condition (The remaining 3-motifs are depicted in Figure 6.)

**Figure 6:**
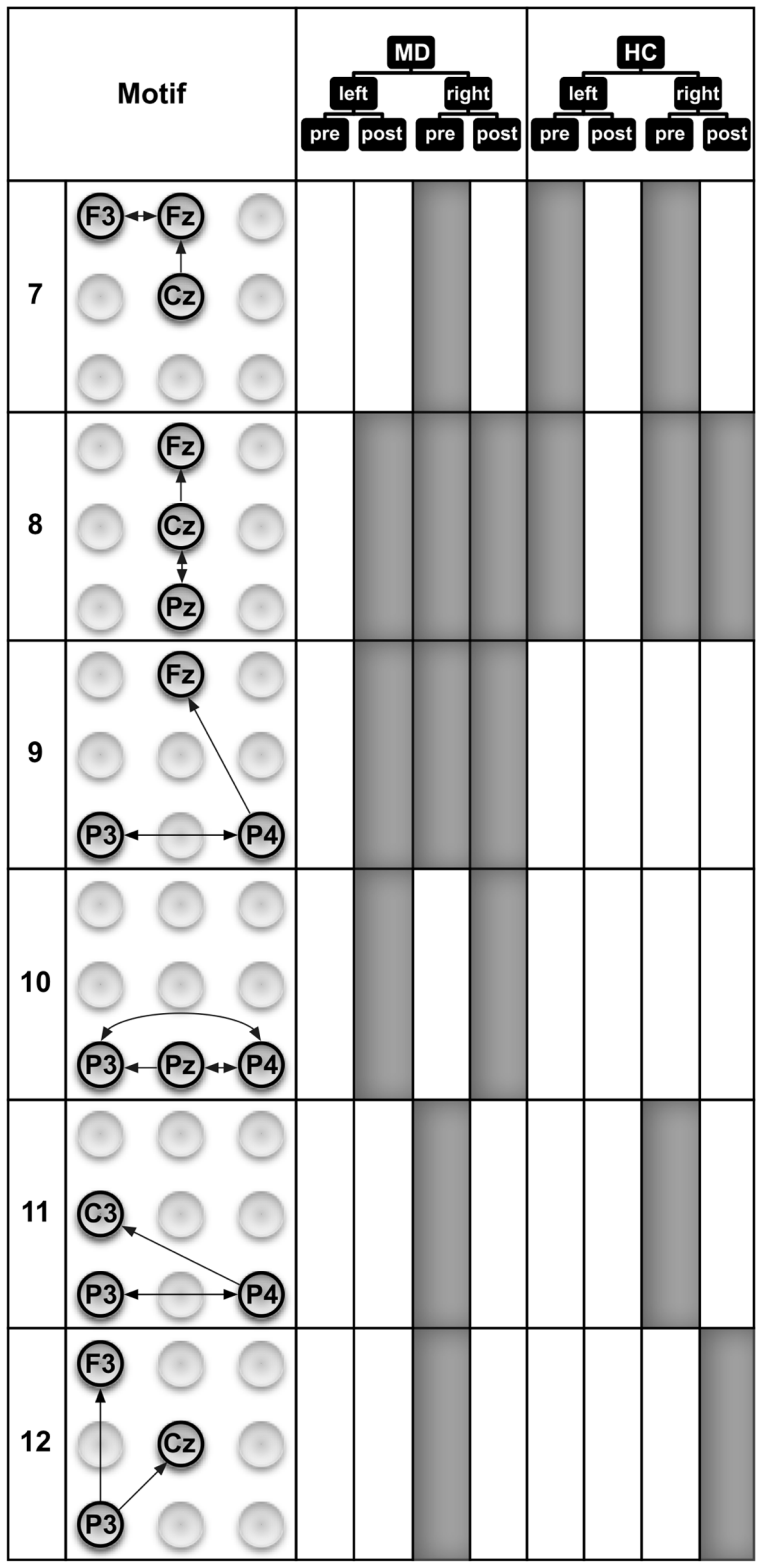
Six out of twelve motifs of size 3 that were detected in the eight ECN samples. The occurrence of a 3-motif in an ECN sample is indicated by filled areas. These motifs represent important directed interaction patterns of brain activity recorded at three different EEG electrodes that occur before and during the processing of painful electrical stimuli. The ECN samples stem from combinations of the group assignment and experimental conditions: MD–patients with major depression, HC–healthy control subjects, left and right–stimulated sides, pre and post–time windows with respect to the stimulus condition (The other 3-motifs are depicted in Figure 5.)

Similarly to motifs of size 2, motifs of size 3 (Figure 5 and Figure 6) are also more often identified in MD than in HC subjects. However, the exact communalities are far less expressed for the motifs of size 3 (5 of 18 in MD; 5 of 13 in HC) compared to size 2 (8 of 12 in MD; 8 of 9 in HC). The results seem to indicate that some motifs in HC are replaced by different motifs in MD. For example, motifs 2 and 3 in HC seem to be replaced by motifs 1 and 2 in MD (including the communality of motif 2 for the processing after stimulation of the right hand). Interestingly, when comparing these motifs between groups, the principle difference lies in stronger activation of the right frontal areas in MD patients. This finding might be interpreted as agreeing with theories on the role of the prefrontal cortex (PFC) in the processing of emotions [51]. The left PFC has been demonstrated to be involved preferentially in processing associated with approach-related, appetitive goals, while the right PFC is more strongly involved in the processing of behavioral inhibition and withdrawal [55], [56]. This theory opens possible interpretations on pathophysiological mechanisms for MD, namely a hypoactivity of the left PFC or a hyperactivity of the right PFC [51]. Our data clearly point to a hyperactivity of the right PFC in our patients. Our data are also consistent with findings indicating the additional recruitment of prefrontal areas by MD patients [57]. While there are nearly as many motifs of size 3 in MD patients during the pre-stimulus period as in the HC subjects, a clear difference can be found in the pre-stimulus period with respect to the site that will become stimulated. Seven out of the 8 motifs of size 3 in MD patients were found before stimulation of the right hand, only one motif was found before stimulation of the left hand. In contrast, in the HC subjects we found 4 motifs of size 3 before stimulation of the right hand and 3 motifs before stimulation of the left hand. Obviously, there is a clear preponderance of motifs before stimulation of the right hand in MD patients. One reason for this preponderance might lie in the contralateral organization of somatosensory information processing. Thus it might be more demanding for MD patients to recruit resources for the analysis of the left hand stimulation because the resources had to be redistributed from the more active right to the left hemisphere. In line with this interpretation, most of the motifs active during the preparation to stimulation of the right hand in MD patients include directed information flow to or within the left hemisphere (i.e., motifs 1, 7, 9, 11, and 12). Another somewhat surprising finding is that there are slightly more motifs in the post-stimulus period found in MD patients. It was previously found that MD patients compared to HC exhibit higher pain thresholds to external stimulation including electrical stimulation [48], [58], lower sensitivity to C-fiber activation [59], and/or lower sensitivity to experimental nociceptive stimulation [58], [60]. However, it should be mentioned that the stimulation was performed with stimuli that were adjusted for subjective pain ratings (i.e., moderately painful in both groups). This might be the reason that there is no obvious difference in the number of motifs found in MD vs. HC subjects. Nevertheless, there are clear differences with respect to the motifs themselves.

Thus there exists an interesting phenomenon regarding differences in motif composition between the MD and HC group after stimulation. It seems that the connectivity due to stimulation shows an opposite direction compared to the pre-stimulus connectivity. With regard to left- and right-hand stimulation, MD showed an equal number of motifs (5), whereas the HC showed more motifs after right-hand stimulation (6) and only one after left-hand stimulation. This might again represent the nature of stimulation with a preferred contralateral processing of the information. So the noxious stimulation of the right hand will primarily (or, at least, more quickly) activate the left hemisphere, but nociceptive processing will activate behavioral inhibition and withdrawal. Thereby a need exists to transfer the information from the left hemisphere to the right and to activate the right PFC due to the noxious stimulation in the HC subjects. Indications for such a transfer might be seen in motifs 2, 4, or 8. In our MD subjects, there is already a clear preponderance of right hemisphere activation, as discussed in the previous paragraph. This might indicate that activation of the right PFC in MD to the same degree as in the HC, does not occur because it is already activated.

## Discussion

The purpose of the null model used in this study was to distinguish inherently regular topological effects from true topological coincidences in the member networks of a sample. The analytical statistical test of our preceding approach [28] was based on incorporation of more simple properties of the input networks into the null model, which accounts for the mean number of edges of the input network sample. The advantage of this statistical testing is that it can be computed efficiently as no random network ensembles must be generated. However, the null model used in this approach is stricter in assigning significance to topological patterns, as it incorporates more topological information of the input network sample. To generate random null model networks we chose the Markov chain Monte Carlo edge-switching algorithm [18], [34], [39], [40], [41] vs. the process of drawing networks from the configuration model ensemble [33], [34], [35], [36], [37], [38]. Using the edge-switching algorithm for network motif detection is recommended by the authors of [34] as there is a good trade-off between speed and accuracy (uniform sampling of random graphs). Similarly to the edge-switching algorithm the configuration model algorithm uses the information of prescribed in-degree and out-degree sequences. One vertex is uniformly and repeatedly chosen from the set of vertices which have not used up all their outgoing edges and one is chosen from the set of vertices that can still accept ingoing edges, to randomly create a directed edge between them. This can be seen as connecting two types of “stubs”: an out-stub of a vertex to an in-stub of another vertex. Then another such pair of vertices is selected randomly and is again connected by a directed edge until all vertices have all their connections with respect to their degree sequence The configuration model approach suffers from the possible introduction of multiple edges and loops into the randomly constructed network, thereby creating multigraphs or pseudographs. Moreover, due to certain bad sequences of random edge selections structural configurations might emerge that would inevitably lead to the introduction of such degenerating edges at some point in the process. Such a degeneration of the resulting random networks is undesirable and should be avoided since it would affect the statistical test for subnetwork significance in networks that are not degenerate themselves. The introduced error is even greater for the smaller and denser networks that we analyze, because in this case degeneration of respective random networks is more likely than in very large networks where in the limit of large network size the density of multi-edges and loops tends to zero [37]. The configuration model approach does not uniformly generate networks since degenerate networks have lower sampling probability (fewer possible stub-pairings) compared to simple networks [37], [38]. Rejections that would be needed to obviate the addition of degenerating edges are unfortunately somewhat problematic on their own. A sampling bias would be introduced if following the discard of a multi-edge an alternative vertex pair is chosen at random from the set of available vertices with free “stubs” [38], [39]. This modified strategy [61] would be equivalent to an extended exploration of the search space in the neighborhood of non-simple partial directed networks. Thus, the final simple network which is generated would not be drawn uniformly from all possible stub-pairings [38]. Such an introduced bias is increased for networks with heavily tailed in-degree and out-degree sequences [39], because of the existence of hub vertices that are prone to obtain more than one edge between them. The algorithm also might be modified to reject partial networks upon introducing a degenerate edge. It then uniformly samples simple networks with prescribed degrees but its acceptance rate is too small to apply to real-world problems [38]. Nevertheless, numerical experiments have revealed that the modified configuration model algorithm that discards degenerating edges and instead selects a new vertex pair can be acceptable in practice despite of its sampling bias [34]. The non-Markov chain Monte Carlo method “go with the winners” [34], [62] applied to the construction of networks with prescribed degree sequences generates statistically correct samples but is too inefficient for our purposes.

Moreover, other types of null model networks could be adopted. We tested the following random graph models to generate sample-specific null model networks: the 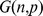 model of Poisson random graphs [16], [37] with probabilities 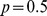 and 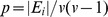, the 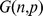 model with the same probabilities as before but with the additional constraint of preserving the number of edges and bidirectional edges of the input network and lastly stickiness index-based networks [63]. We observed that using these other types of null model networks results in a much greater number of unwanted characteristic topological patterns showing inexplicable cross-linkages between the left and right hemisphere. Fewer such cross-linkages are obtained using the edge-switching algorithm for generating null model random networks.

From a computational resources point of view our approach is best suited for smaller networks that typically arise in studies based on EEG recordings (network size 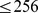 vertices) and moderate network sample sizes. Much computation time can be saved by not determining the parameter values for the network randomization procedure but instead relying on fixed values of (e.g. 100) and a smaller number of null model networks for each input network (e.g. 1000). This, of course, comes at the expense of accuracy.

The concept of gaining understanding of networks and their design principles by decomposition into smaller relevant subunits is accepted, yet it remains controversial. In particular controversy remains focused on the functional interpretation of network motifs and therefore also of locatable characteristic interaction patterns. A null model network needs to incorporate basic structural properties of a network as well as account for the rules that govern its formation to prevent statistical tests from falsely assigning significance to its features or substructures [30], [31]. Otherwise, those properties would incorrectly be more prominent in the statistical test although they are not outstanding features for the examined network. The process by which a network was formed, even if free of selection for or against particular structural patterns that perform functions, can also favor the emergence of significant structures, like network motifs, although they do not necessarily contribute to functionality in the network [30], [31]. Generally, these artifacts in the network motif signature are difficult to distinguish from functional relevant network motifs and sometimes these differences might even be imprecise [31]. Spatial clustering of vertices either in topological space or in attribute space is related to constraints on network construction. A test for whether network motifs might arise solely from these geometric constraints and not as a consequence of additional functional optimization has been proposed [64]. It has been concluded that network motifs in real-world networks are not solely determined by geometric constraints [64], [65]. Whether structure-function relationships might be ambiguous and depend on the structural context in which the subnetworks are embedded in the network has been debated [66]. For a more definite interpretation of motif function it might be necessary to complement topological information with information on parameters that describe dynamical behavior [67]. The primary challenge in future research is to overcome these sorts of complexities. Overall, strengthening the assigned significance of network structures by experiments that reveal whether and how they contribute to network functionality should be a goal of future research, although carrying out such experiments is not an easy proposition. Yet certain network motifs have been tested experimentally and their presumed regulatory roles have been confirmed in bacteria and yeast transcription networks [68], [69].

We use relative frequencies of subnetwork counts in the random ensemble to compute p-values for the respective subnetwork counts in the input ECN sample. In contrast, the use of z-scores is common in other studies [18], [21], [24], [31], making the unsafe assumption that subnetwork occurrences are normally distributed [70], which is not always the case [71].

As a final remark, our method was applied to detect characteristic topological patterns in networks derived from a connectivity analysis in the sensor space. Therefore, stringent informative value with respect to anatomical locations of those patterns is limited.

## Supporting Information

Materials S1 Supplementary Material that contains further information on the EEG experiments, the connectivity analysis and a characterization of the resulting network samples that are the data basis for this study.
(PDF)
