# Supplementary material for: Extracting Labeled Topological Patterns from Samples of Networks
Source: PLoS One. 2013 Aug 12;8(8):e70497. doi: 10.1371/journal.pone.0070497 (PMC3741309; doi:10.1371/journal.pone.0070497)
Supplement: Materials S1 — Supplementary Material that contains further information on the EEG experiments, the connectivity analysis and a characterization of the resulting network samples that are the data basis for this study. (PDF) [file pone.0070497.s001.pdf]

Supplementary Material

-Obtaining Locatable Characteristic Topological Patterns from Samples of Effective Connectivity Networks with Fixed Pairwise Different Vertex Labels-

Materials and Methods

Subjects

Eighteen patients (10 women, 8 men) with major depression (mean age $\pm$ standard deviation: 38.9 $\pm$ 15.5 years) and 18 sex- and age-matched healthy control subjects (39.3 $\pm$ 14.8 years) participated in this study. Major depression was established according to DSM IV criteria using a structured interview, and the Beck depression inventory (BDI) was also administered. BDI scores of patients ranged from 19 to 48 (29.4 $\pm$ 9.7); scores of control subjects were all below five (2.1 $\pm$ 1.5). All subjects were right-handed. Nine patients were treated with antidepressant medication (5 patients received selective serotonin reuptake inhibitors, SSRIs; 4 patients norepinephrine and serotonin reuptake inhibitors, NaSRIs) while the remaining participants did not receive any medication. One patient and two controls had to be excluded from the study because it was not possible to determine meaningful individual stimulus-response properties to intracutaneous electrical stimuli. Prior to the experiment detailed information on the aim and the procedures of the experiment was provided to each subject and written informed consent was obtained. The procedure was approved by the Ethics Committee of the Friedrich Schiller University (reference number 2282-04/08).

EEG recording and connectivity analysis

All subjects received intracutaneous electrical stimulation at the tip of the middle fingers of both the right and the left hand. The current intensity level was adjusted between 10 μA and 1 mA. Stimuli consisted of a bipolar rectangular pulse of 10 ms duration. Participants were requested to rate each

electrical stimulus on a scale ranging from 0 to 6 (0 - no sensation; 1 - just perceived, not painful; 2 - clearly perceived, but not painful; 3 - low pain; 4 - moderate pain; 5 - strong pain, but tolerable; 6 - unbearable pain) [1,2] . The pain threshold was defined as the intensity yielding a sensation described as a sharp painful pinprick, corresponding to a rating of "3". The EEG was recorded continuously during the electrical stimulation from 60 electrodes, referenced to Cz, using a standard EEG cap (Easy Cap, Falk Minow Services, Germany) based on an extended International 10-20 system. Finally, data from nine selected electrodes F3, Fz, F4, C3, Cz, C4, P3, Pz and P4 (re-referenced to a linked ears reference) were processed. These electrodes are situated above some of the important regions of pain processing, attention and depression (frontal, central, and parietal brain regions). Eye movement and mastication muscle activity artifact contaminated single trial somatosensory evoked potentials (SEPs) were excluded, which resulted in an exclusion of three data sets since there were not enough artifact-free trials left for a reliable connectivity analysis. In order to compare the pre- and post-stimulus condition, signal sections of 700 ms duration were extracted pre-stimulus onset ($700\ ms$ before onset to the onset of stimulus, i.e. $-700\ ms$ to $0\ ms$) as well as post-stimulus onset (from stimulus onset to $700\ ms$ after stimulus onset, i.e. $0\ ms$ to $700\ ms$). These signal sections provided the data basis for the connectivity analysis. To assess the effective connectivity between each ordered pair of nine electrodes, the generalized partial directed coherence (gPDC) [3] was applied. The frequency range of interest for the SEP analysis was determined to be in the delta-, theta- and the alpha-bands (1 to 13 Hz) since the signal power is mainly situated in this frequency range. For a consolidated analysis the gPDC values of the corresponding frequencies were pooled to one quantity by averaging with respect to the frequency range of interest. Thus, one gPDC value results for each of the 72 possible directed interactions. Finally, the effective connectivity that we are interested in is given by significantly increased gPDC values. The significance test was conducted by the Bootstrap procedure introduced in [4] at a significance level of 5%. In the present study, we generally used 1500 Bootstrap repetitions. A drawback of this approach might be that the gPDC distribution under the null hypothesis ‘no interaction’ cannot be guaranteed. In this case, the significance threshold cannot be determined. With respect to the entire sample, we detected this situation in 3.48% of all possible directed interactions. We registered and treated the connections as missing values in these cases. By treating significant gPDC values as existent interactions of the respective subject, we modeled each subject’s EEG recorded neural activity that occurred over the period of the corresponding stimulus condition as an ECN.

Imputations of missing values

Connectivity analysis is complicated by the fact that the significance threshold cannot be determined in all cases. Even though this situation rarely appears, many networks are partially unobserved. In particular, only 12.5% of the networks are not affected by at least one missing value. As a consequence,

traditional analysis (listwise deletion of networks with missing values) would discard a huge amount of useful information of the data. Besides this, excluding networks implies that the remaining networks are a random subsample of the original data set (i.e. the data are missing completely at random (MCAR)). This is clearly a very strong assumption and it is likely that it is not fulfilled for our data. Therefore, estimates and inferences derived from only our fully observed networks would be biased. In the literature, different imputation strategies exist to overcome the problem of missing values [5]. Since there is no superior approach concerning all possible missing data mechanisms, a reasonable imputation strategy according to the specific data set has to be chosen.

Because it seems implausible that observed variables contain useful information to predict missing values (i.e. data are missing at random (MAR)), we performed extreme case imputation: First, all missing directed edges are imputed as no interactions, while in the second data set they are treated as interactions. Both data sets are analyzed independently, and only subnetworks that are significant in both cases are considered as locatable characteristic topological patterns. This is a conservative approach to deal with the problem of missing values, and it keeps the effect of the imputations on topological pattern selection as small as possible.

Effective connectivity networks

Effective connectivity networks are directed graphs, which have identical pairwise different vertex labels. They consist of a non-empty finite set V of vertices –the EEG channels– and a finite set E of ordered pairs of distinct vertices called arcs or directed edges, which represent directed interactions between each electrode pair. Every vertex is associated with the position of its recording EEG-electrode, which becomes its label. The ordered pair (v_i, v_j) denotes a directed edge that leaves vertex v_i and enters vertex v_j. The vertex v_i is called the tail and vertex $v_j$the head of the edge. Each directed edge is given by a corresponding significant gPDC value. An ECN might be represented by its adjacency matrix A. Since we computed effective connectivity between signals from nine selected electrodes, each ECN has a vertex set of cardinality $|V| = 9$ and the adjacency matrices A have size 9×9 where $A_{ij} = 1$ if and only if the ECN comprises the directed edge (v_i, v_j). Accordingly, a bidirectional edge running between two vertices v_i and v_j is indicated by two entries $A_{ij} = 1$ and $A_{ji} = 1$ in A. Most ECNs have dense connection patterns. The mean number of directed edges in our samples of ECNs is 36.8 out of 72 potential edges. Since ECNs are simple directed graphs they do not contain loops (edges whose tail and head coincide) or multiple edges (multi-edges – edges that have the same tail and the same head). With two exceptions, all ECNs are connected networks.

In our study we examined eight samples of ECNs. These eight samples result from all combinations of the group assignment (MD – patients with major depression, HC – healthy control subjects), the time window with respect to the stimulus condition (pre – time window before stimulation, post – time window following stimulation) and the stimulated side (right – right hand, left – left hand). The size for the “MD-post-right” sample is fifteen; where for all other samples the size equals sixteen.

Estimation of an upper bound for the number of networks with prescribed degree sequence

We obtained a lower bound for the number of pairwise different networks with the prescribed degree sequence of the representative ECN by means of simulations. To put it into perspective we were also interested in estimating an upper bound. Since it has 37 directed edges, obviously, there cannot exist more than 2^{72} square matrices of size nine. An only slightly better estimation for the number of such networks is given by $\binom{72}{37}$. An appropriate decomposition of the adjacency matrix of the representative ECN yields 131,712,000 as a much better upper bound for the number of matrices with same row and column sums as the adjacency matrix of the representative ECN. The adjacency matrix is binary and its diagonal elements are not considered in the decomposition. Let k_i be the sum of the i-th row and let l_i be the sum of the i-th column. For the first row there are $\binom{8}{k_1}$ combinations of positions for the one-entries (edges). Since each such placement affects the number of ways to place one-entries in the second row, there are only $\binom{7}{k_2-A_{21}}$ combinations possible. For the third row there are $\binom{6}{k_3-A_{31}-A_{32}}$ combinations of one-entries. For the fourth row there are $\binom{5}{k_4-A_{41}-A_{42}-A_{43}}$ such combinations. Similarly one can calculate the combinations of positions for the one-entries in the columns of the matrix. These combinations for the first, second, third and fourth column are given by $\binom{8}{l_1}$, $\binom{7}{l_2-A_{12}}$, $\binom{6}{l_3-A_{13}-A_{23}}$ and $\binom{5}{l_4-A_{14}-A_{24}-A_{34}}$, respectively. The value for the upper bound for the number of networks with the same in-degree and out-degree sequence as the representative ECN is then yielded by multiplication of these combinations

$$\binom{8}{k_1} * \binom{7}{k_2-A_{21}} * \binom{6}{k_3-A_{31}-A_{32}} * \binom{5}{k_4-A_{41}-A_{42}-A_{43}} * \ldots * \binom{8}{l_1} * \binom{7}{l_2-A_{12}} * \binom{6}{l_3-A_{13}-A_{23}} * \binom{5}{l_4-A_{14}-A_{24}-A_{34}} * \ldots * \binom{1}{l_8-A_{18}-A_{28}-A_{38}-A_{48}-A_{58}-A_{68}-A_{78}} = 131{,}712{,}000$$

Binomial coefficients $\binom{n}{k}$ with negative k were ignored in these calculations.

References

1. Meissner W, Weiss T, Trippe RH, Hecht H, Krapp C, et al. (2004) Acupuncture Decreases Somatosensory Evoked Potential Amplitudes to Noxious Stimuli in Anesthetized Volunteers. Anesthesia & Analgesia 98: 141-147.
2. Weiss T, Kumpf K, Ehrhardt J, Gutberlet I, Miltner WHR (1997) A bioadaptive approach for experimental pain research in humans using laser-evoked brain potentials. Neuroscience Letters 227: 95-98.
3. Baccala LA, Sameshima K, Takahashi DY. Generalized partial directed coherence; 2007. IEEE. pp. 163-166.
4. Sato JR, Takahashi DY, Arcuri SM, Sameshima K, Morettin PA, et al. (2009) Frequency domain connectivity identification: An application of partial directed coherence in fMRI. Human Brain Mapping 30: 452-461.
5. Enders CK (2010) Applied Missing Data Analysis: Guilford Press. 1-377 p.
